# *Candida auris* Outbreak and Epidemiologic Response in Burn Intensive Care Unit, Illinois, USA, 2021–2023

**DOI:** 10.3201/eid3103.241195

**Published:** 2025-03

**Authors:** Hannah J. Barbian, Louise Lie, Alyse Kittner, Amanda Harrington, Joshua Carson, Mabel Frias, David H. Slade, Do Young Kim, Stephanie Black, Jorge P. Parada, Mary K. Hayden

**Affiliations:** Rush University Medical Center, Chicago, Illinois, USA (H.J. Barbian, M.K. Hayden); Loyola University Medical Center, Maywood, Illinois, USA (L. Lie, A. Harrington, J. Carson, D.H. Slade, J.P. Parada); Chicago Department of Public Health, Chicago (A. Kittner, D.Y. Kim, S. Black); Cook County Department of Public Health, Forest Park, Illinois, USA (M. Frias)

**Keywords:** Candida auris, fungi, disease outbreak, intensive care unit, genomics, whole-genome sequencing, phylogeny, disinfection, hand hygiene, antimicrobial resistance, Chicago, Illinois, United States

## Abstract

*Candida auris* is an emerging fungal pathogen associated with outbreaks in healthcare settings. We report a multiyear outbreak of *C. auris* in a burn intensive care unit in Illinois, USA, during 2021–2023. We identified 28 *C. auris* cases in the unit over a 2-year period, despite outbreak response and multimodal mitigation measures. Of the 28 case-patients, 15 (53.6%) were considered colonized and 13 (46.4%) had clinical infections. Phylogenetic analysis of whole-genome sequences revealed 4 distinct clusters of closely related (0–6 SNP differences) genomes containing 3–6 cases. Clusters generally contained temporally related isolates from patients with epidemiologic links; this finding suggests that multiple introductions and within-unit spread over a limited time were responsible for the outbreak, rather than transmission from a long-term source (e.g., persistent environmental contamination or staff carriage). Here, integrated traditional and genomic epidemiology supported *C. auris* outbreak investigation and response and informed targeted interventions.

*Candida auris* is a fungal pathogen associated with colonization and high-mortality invasive infections in persons with underlying medical conditions, especially those who are hospitalized or reside in long-term care facilities (*1*,*2*). Prolonged skin colonization and environmental contamination likely contribute to within-facility persistence and spread (*2*–*5*). *C. auris* often displays extensive antifungal resistance and can acquire resistance rapidly during antifungal treatment (*6*–*8*).

Intensive care units (ICUs) are particularly vulnerable to *C. auris* outbreaks because of prolonged patient stays, high medical acuity, and extensive use of medical devices that can encourage pathogen spread (*9*–*12*). Effective infection prevention strategies are key to curbing the spread of *C. auris*; those strategies include contact screening, strict hand hygiene procedures, appropriate use of personal protective equipment (PPE) and transmission-based precaution by healthcare providers, use of single-patient equipment, environmental cleaning and disinfection, and private-room isolation (*13*). However, *C. auris* colonization and transmission have been reported to persist despite aggressive infection prevention interventions, making *C. auris* control a long-term burden in affected facilities (*12*,*14*,*15*).

In burn ICUs (BICUs), patients are at increased risk for healthcare-acquired infections because of breakdown of the skin barrier and the immunocompromising effects of burns; infection is the leading cause of death after burn injury (*16*). Fungal wound infections are reported in 6%–45% of all burn admissions; candidemia develops in up to 5% of patients with severe burns. Unlike most *Candida* species, *C. auris* has a tropism for skin (*17*), and it can readily colonize or infect adjacent large, open, nutrient-rich burn wounds. Furthermore, because they have frequent infections and large, open wounds, burn patients often require treatment with systemic and topical antimicrobials, both of which have capacity to eliminate competitive microbiota and encourage colonization with resistant organisms such as *C. auris.* Care provided in BICUs, such as skin debridement, may disperse colonized or infected skin cells into the environment, which contributes to transmission.

We describe a *C. auris* outbreak and response in a BICU in Illinois beginning in 2021. We used whole-genome sequencing (WGS) to help refine epidemiologic inferences and direct interventions. WGS has been used to support epidemiologic investigations of *C. auris* infection, including hospital outbreaks (*9*,*18*–*23*). Outbreak sequences generally form a unique clade with limited diversity (*9*,*18*); close relationships have been observed between epidemiologically linked cases (median 7 SNPs) and isolates from the same person (median 2 SNPs) (*21*). WGS can also detect antifungal resistance mutations (*19*,*24*,*25*). Thus, WGS may be a powerful tool to support *C. auris* outbreak investigations.

## Methods

### Study Setting and Participants

The Burn Center is a 10-bed intensive care unit caring for pediatric and adult burn patients at a 547-bed academic tertiary care medical center in the Chicago metropolitan area, Illinois, USA. The unit accommodates ICU overflow from other services, including medical and surgical ICUs. The unit practices universal contact precautions (gowns, gloves, masks, and eye protection) for all patients, staff, and visitors to the unit. We abstracted patient data via retrospective review of the hospital electronic medical records.

The Institutional Review Board of Loyola University (Chicago, IL, USA) reviewed and approved the protocol for this study (LU218571). Informed consent was waived.

### Case Identification and Investigation

The outbreak investigation, led by the infection prevention team, consisted of admission screening and weekly point prevalence surveys of all patients in the unit. We defined a hospital-acquired case of *C. auris* as any illness in patient who, after a negative *C. auris* admission screen, tested positive for *C. auris* on subsequent weekly point prevalence screens or in any clinical specimen. We defined colonized cases as patients who had *C. auris* identified from surveillance cultures but no detection of *C. auris* in any clinical specimens. Clinical cultures refer to blood, wound, respiratory, or urine cultures.

We conducted epidemiologic investigations to identify commonalities between cases, including healthcare workers, medical equipment, prior room occupancies, and exposure locations outside of the BICU, including the operating room, tub room, and procedural areas such as the interventional radiology and gastroenterology suites. We reviewed patients’ history of *C. auris* through query of the Illinois extensively drug-resistant organism registry (*34*).

### Infection Control Measures

Universal contact precautions and masks are used for all BICU patients; further containment strategies implemented in response to this outbreak involved increased observation of isolation compliance, education of nursing and ancillary staff about *C. auris* transmission and control, environmental cleaning validation, enhanced environmental cleaning with ultraviolet (UV) light, observation and training regarding correct use of PPE, and proper hand hygiene. In addition, the local health department performed an infection control assessment and response to identify and address infection control gaps.

### Microbiologic Identification of Cases

We isolated *C. auris* fungus from screening samples collected from the axilla and groin of patients using a BBL CultureSwab EZ Collection and Transport System (BD, https://www.bd.com). We then inoculated samples onto HardyCHROM Candida (Hardy Diagnostics, https://hardydiagnostics.com) and incubated aerobically, protected from light, at 35°C for 72 hours. We isolated *C. auris* from clinical samples submitted for routine diagnostic testing on standard microbiologic media including sheep blood agar, chocolate agar, inhibitory mold agar (BBL prepared plated media; BD), and blood culture media (BACTEC Plus Aerobic and Lytic Anaerobic media; BD). We performed species identification by using Biotyper matrix-assisted laser desorption/ionization time-of-flight (MALDI-TOF) mass spectrometry with the MBT Compass Library version 12 Revision K (Bruker Daltonics, https://bruker.com).

### Whole-Genome Sequencing

We suspended available *C. auris* isolates in DNA/RNAshield (Zymo, https://zymoresearch.com) and transported them to the Regional Innovative Public Health Laboratory at Rush University Medical Center (Chicago, IL, USA). We extracted nucleic acids using the Cultured Cells DNA Kit and Maxwell extraction system (Promega, https://promega.com) and prepared sequencing libraries using 1 ng DNA extract and Nextera XT DNA Library Preparation Kit (Illumina, https://illumina.com). We barcoded genome libraries by using IDT for Illumina DNA/RNA UD Indexes (Illumina) and balanced using a small-scale sequencing run of an equivolume pool (Illumina iSeq). We subjected final libraries to 2 × 150 paired-end sequencing on NovaSeq6000 (Illumina). We submitted data to the National Center for Biotechnology Information Short Read Archive (Appendix Table 1).

### Bioinformatic and Statistical Analysis

We downloaded all publicly available Illinois *C. auris* sequences for comparison to outbreak sequences (Appendix Table 1). We analyzed paired-end sequences with the MycoSNP-nf pipeline version 1.4 (https://github.com/CDCgov/mycosnp-nf) by using clade IV reference B11243 (Genbank accession no. GCA_003014415.1) and implemented on Terra as previously described (*20*,*27**,**28*), excluding isolates with estimated coverage depth <25. We determined *C. auris* clade using phylogenetics with clade I–IV reference sequences. We used SNP differences between all samples and the reference to build a neighbor-joining tree using MEGA 11 (*29*) as previously described with 1,000 bootstrap replicates (*21*,*30*). To compare SNP differences, we used SNP distance matrices with all available Illinois sequences. To identify potential antifungal-resistance mutations, we used Snippy (*31*) to query for mutations in the *FKS1, ERG11, TAC1b, MRR1, ERG3,* and *FUR1* genes. We compared mean SNP differences using Kruskal-Wallis nonparametric testing with adjusted significance between individual groups calculated using the Dunn multiple comparisons test.

## Results

### Outbreak Investigation

**Figure 1:**
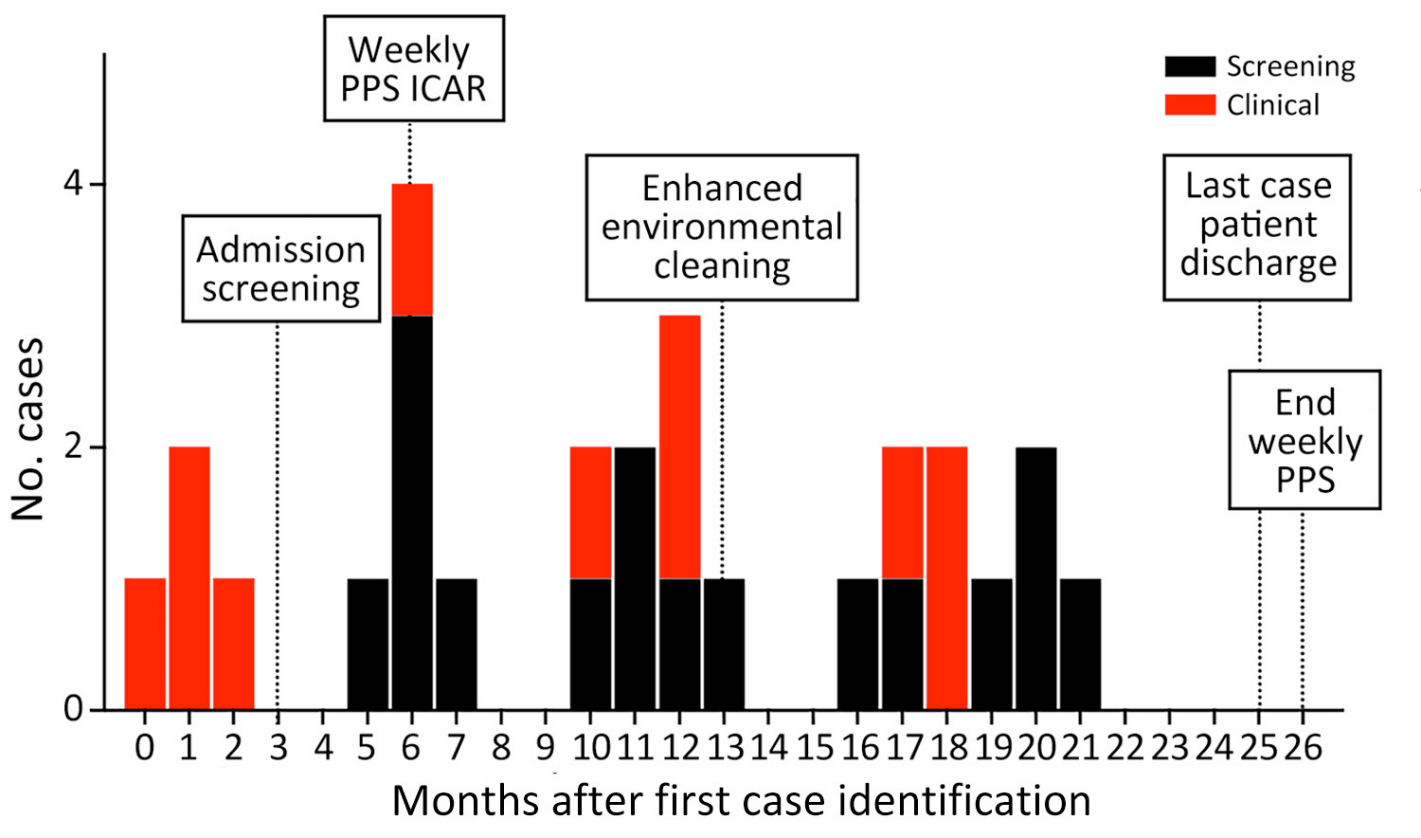
Epidemiologic curve of *Candida auris* outbreak cases in burn intensive care unit, Illinois, USA, 2021–2023. Color indicates whether case was identified by screening or clinical isolates. PPS, point prevalence survey.

The first clinical *C. auris* isolate in a BICU patient was identified from blood culture in 2021. Three additional case-patients with hospital-acquired *C. auris* were identified in the subsequent 2 months (Figure 1). Admission screening in the BICU was initiated 3 months after the first case-patient was detected (Figure 1). A fifth case-patient, who originally screened negative on admission, was identified from a wound culture 83 days after admission; that case was notable because it was the first confirmed hospital acquisition of *C. auris*. A point prevalence survey 5 months after first case detection identified a sixth case. Weekly point prevalence screening was initiated 6 months after first case detection; 22 additional cases were identified 6–21 months after first case identification (Figure 1). Weekly point prevalence surveys were discontinued 28 days after discharge of the last patient with *C. auris*.

We reviewed case records to identify documented epidemiologic links; specifically, common locations (rooms), procedures, and staff exposures. Intensive observation of infection control practices throughout the BICU identified breaches that may have contributed to *C. auris* transmission, including poor hand hygiene compliance, improper PPE donning and doffing, cluttered patient care areas preventing thorough environmental cleaning, poor auditing of environmental cleaning, and inconsistent cleaning and disinfection practices for shared equipment. Shared equipment within the unit included bladder scanners, forced-air patient warming devices, vascular Dopplers, EKG machines, point-of-care ultrasounds, and recliners. Particular attention was given to staff whose patient care activities were extended to areas in the medical center outside of the BICU, including physical, occupational, speech and respiratory therapy, and radiology staff. The hospital infection control team and the local health department observed infection control breaches during the infection control assessment performed 6 months after the first case of *C. auris* was detected.

### Outbreak Mitigation Measures

Early in the outbreak, a multidisciplinary *C. auris* response team, including staff from infection prevention, environmental services, nursing, facilities management, BICU physicians and hospital leadership, convened to create and implement a structured plan. All patients in the BICU with positive *C. auris* culture results were placed on contact precautions; signs were placed on the patients’ room doors, and their electronic medical records were flagged for *C. auris* and an isolation order. The team implemented outbreak mitigation measures universally in the BICU and centered on communication, education, and process improvement, focusing on environmental cleaning and hand hygiene. Education on *C. auris* transmission and necessary precautions were extended to the BICU nursing staff, with special attention on ancillary groups, particularly those also providing care to units outside of the BICU: environmental service, respiratory therapy, physical and occupational therapy, food and nutrition services, radiology, and pastoral care.

The team reviewed cleaning responsibilities between nursing and environmental service, including method of cleaning and frequency. Standard cleaning practices include floor and surface cleaning with a disinfectant effective against *C. auris,* bleach-wipe cleaning of equipment, and a log to track cleaning of shared equipment. Storage cabinets were installed in patient rooms. Black-light audits on discharge cleans were required on every terminal discharge to monitor cleaning practices, and environmental service staff received coaching when cleaning failures were identified. Germicidal ultraviolet disinfection was performed in patient rooms and above the unit’s nursing station beginning 9 months after first case detection. Thirteen months after first case detection, terminal cleaning of patient rooms incorporated high-intensity UV disinfection.

Unobtrusive-observer audits revealed that overall hand hygiene compliance was 78%–93% during the outbreak period. Most observations were of nursing staff; the greatest opportunities for improvement in compliance were among patient transporters (32% compliance), food and nutrition services (35% compliance), and physicians (67% compliance). The team increased hand hygiene promotion signage and efforts to normalize just-in-time coaching for hand hygiene and PPE breaches among staff and visitors.

### Patient Characteristics

**Figure 2:**
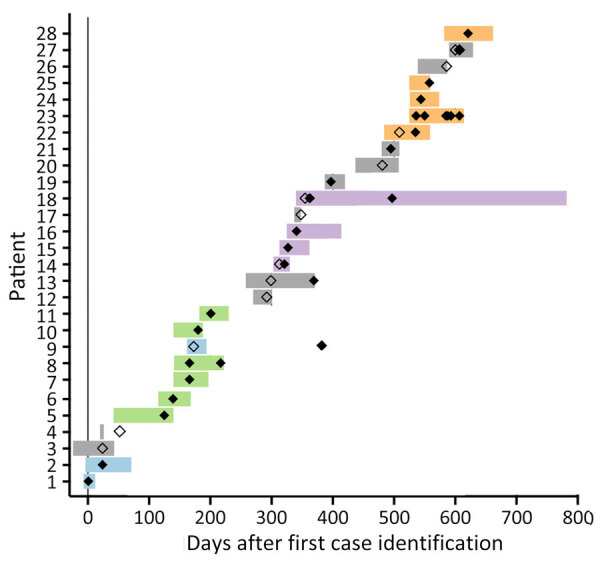
Case timeline for outbreak of *Candida auris* in burn intensive care unit (BICU), Illinois, USA, 2021–2023. Horizontal bars indicate BICU admission duration for each patient; bar colors indicate genomic cluster (blue, cluster 1; green, cluster 2; purple, cluster 3; orange, cluster 4; gray, not sequenced or no cluster). Diamonds indicate collection date of the first *C. auris* isolate and any subsequent isolates that were subjected to whole-genome sequencing; filled diamonds mean the isolate was sequenced, unfilled, not sequenced. In 2 instances (patient 4 and 9), *C. auris* was isolated after discharge from the BICU.

During the 21-month investigation, 28 patients were colonized or infected with *C. auris* (Table); 4 patients had invasive *C. auris* before admission screening. The average patient age was 49 years (range 16–81 years). Most patients were admitted with burns (64%), 9 patients (32%) were admitted with soft-tissue infections, and 1 patient was on medical ICU service. None of the patients had a history of *C. auris* infection, determined by chart review and query of the Illinois extensively drug-resistant organism registry. Seven patients were admitted from outside hospitals or had a hospitalization <30 days before the BICU admission; none were admitted from skilled nursing facilities. The mean length of stay in the BICU before identification of *C. auris* was 26 days (range 7–83 days). *C. auris* was identified in clinical cultures from 13 patients, some of which had *C. auris* in multiple cultures; 8 had *C. auris* identified in blood culture, 6 in respiratory culture, 8 in wound culture, and 3 in urine culture. The mean total length of stay in the BICU was 67 days (Figure 2).

**Table:** Characteristics of 28 *Candida auris* outbreak case-patients in BICU, Illinois, USA, 2021–2023*

Characteristic	Value
Sex	
F	13 (46)
M	15 (54)
Average age, y (range)	49 (16–81)
Admission diagnosis	
Burn	18 (64)
Soft tissue infection not including burns	9 (32)
COVID-19†	1 (4)
*C. auris* culture source	
Axillary/inguinal screening culture‡	24 (86)
Clinical culture§	14 (50)
Blood	8 (29)
Respiratory	6 (21)
Wound	8 (29)
Urine	3 (11)
Co-infection with multidrug-resistant organism¶	13 (46)
Mean length of stay from admission to first positive *C. auris* culture, d (range)	26 (7–83)
Recent hospitalization <1 month before hospitalization	8 (29)
Medical devices used <1 week before positive *C. auris* culture	
Central venous catheter	24 (86)
Ventilator	18 (64)
Urinary catheter	24 (86)
Ancillary medical services received <1 week before first positive *C. auris* culture	
Occupational therapy	23 (82)
Physical therapy	18 (64)
Speech therapy	5 (18)
Mean length of stay in BICU, d (range)	67 (6–442)
*C. auris* outcome	
Colonization	14 (50)
Infection	13 (46)
Discharge disposition	
Skilled nursing facility, acute rehab or other hospital	17 (61)
Home	5 (18)
Deceased	6 (21)

*Values are no. (%) except as indicated. BICU, burn intensive care unit. †Medical intensive care unit service patient on overflow to BICU. ‡Ten of the 24 patients with a positive axillary/inguinal screen also had *C. auris* identified from a clinical culture. §Of the clinical cultures, 4 patients had *C. auris* identified only in blood, 3 had *C. auris* only from wound infections, 1 had *C. auris* only from respiratory source, and the remaining 6 had *C. auris* identified in >1 clinical culture source. ¶A total of 13 patients were co-infected or co-colonized with 19 other multidrug-resistant organisms: 6 vancomycin-resistant enterococci, 5 extended-spectrum β-lactamase–producing organisms, 3 methicillin-*resistant Staphylococcus aureus*, 2 multidrug-resistant *Pseudomonas aeruginosa* (MDR-PA), 1 carbapenem-resistant *Acinetobacter baumannii*, 1 carbapenem-resistant Enterobacterales, 1 AmpC β-lactamase–producing organism.

### Genomic Analysis of Outbreak Isolates

To investigate the genetic relationship of *C. auris* among cases, we conducted WGS on available isolates from the BICU outbreak, including isolates from 22 (79%) of 28 case-patients and 8 longitudinal isolates from 3 of those patients (Figure 2). Isolates from the remaining 6 case-patients were not available for analysis. We also sequenced available contemporaneous isolates from the same facility (n = 19) or another healthcare facility within the medical system (n = 31) to estimate diversity of hospital *C. auris* isolates and identify potential links outside of BICU. Of 80 isolates, 78 (97.5%) had sufficient genome quality for analysis; all were *C. auris* clade IV. Comparison with publicly available *C. auris* sequences from Illinois (n = 364) revealed that genomes from the BICU and related facilities were interspersed throughout other Illinois *C. auris* sequences (Appendix Figure 1), indicating multiple transmissions to the BICU facility from the broader diversity in the region.

**Figure 3:**
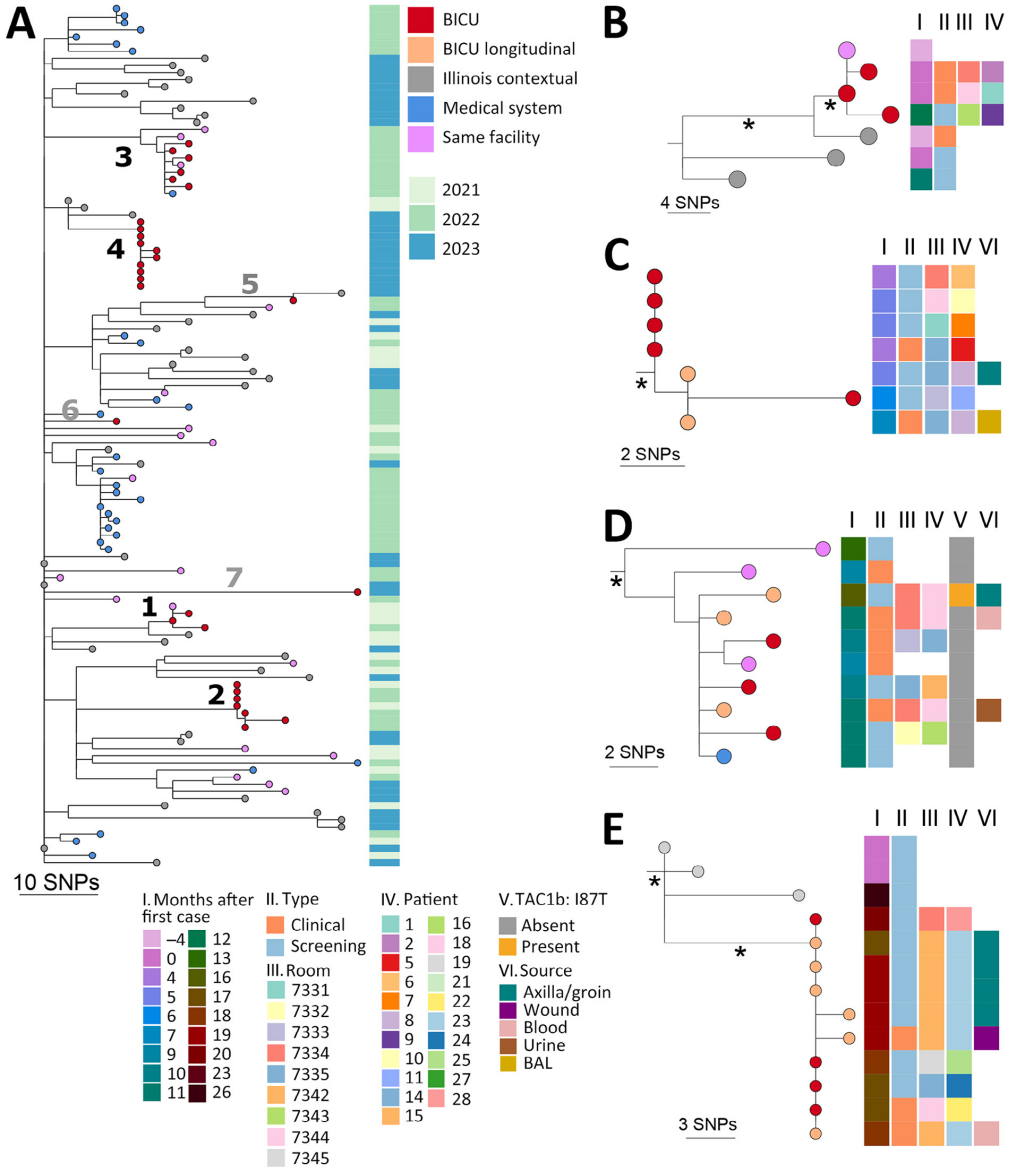
Genomic analysis of outbreak and contemporaneous contextual *Candida auris* isolates in outbreak of *C. auris* in BICU, Illinois, USA, 2021–2023. A) Neighbor-joining SNP-based phylogenetic tree of sequences from BICU isolates, isolates collected from the same facility or another facility within the medical system, and publicly available Illinois sequences collected in 2021–2023. Facility source for 31 of 43 Illinois contextual sequences was confirmed as not within the BICU medical system. The facility source of the remaining 12 isolate sequences was not known. Branch lengths are SNP distances. Isolate collection year is indicated in metadata column to the right. Numbers 1–4 indicate branches leading to BICU clusters; numbers 5–7 indicate branches leading to BICU isolates that do not cluster with others. B–E) Subtrees from BICU cluster 1 (B), cluster 2 (C), cluster 3 (D), and cluster 4 (E). Relevant isolate and patient metadata are indicated in the columns to the right of tree tips; key at bottom shows metadata coding for panels B–E. Orange tips indicate isolates collected from the same person. Isolate collection date is shown as months after the first BICU case. Asterisks (*) indicate branches with >95% bootstrap support. Scale bars indicate SNPs. BICU, burn intensive care unit; SNP, single-nucleotide polymorphism.

**Figure 4:**
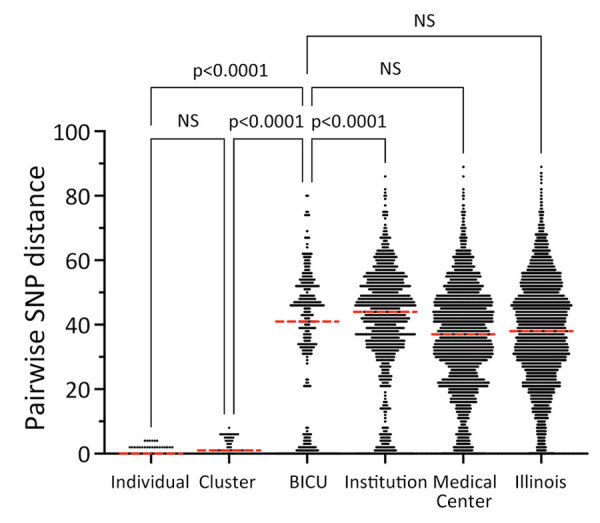
Pairwise SNP distances within different *Candida auris* populations in study of outbreak in burn intensive care unit, Illinois, USA, 2021–2023. Black points (which appear as lines for the large datasets) are pairwise SNP distances between 2 isolate sequences; horizontal red dashed lines indicate medians. BICU, burn intensive care unit; NS, not significant (adjusted p>0.05); SNP, single-nucleotide polymorphism.

BICU isolates formed 4 clusters within Illinois sequences (Figure 3, panel A). Clusters contained 3–6 unique patients and closely related genomes (0–8 SNP differences) (Figure 3, panels B-E). Three BICU isolates did not cluster closely with any other BICU or Illinois isolate, differing by 7–39 SNPs from the closest non-BICU and 21–49 SNPs from the closest BICU isolate (Figure 3, panel A; Appendix Figure 1). We identified a mean 1.9 (range 0–8) SNP differences within BICU clusters, which was not significantly different from SNP differences from isolates collected from the same patient (p>0.9999) (Figure 4). However, SNP differences among all BICU isolates were significantly higher (mean 34.6; p<0.0001), but not significantly different from, mean SNP differences among isolates collected within the same timeframe elsewhere within the medical center (mean 35.8) or all Illinois (mean 37.9). Those findings indicate that BICU clusters were very closely related among isolates within the cluster but not more closely related among clusters than for other regional isolates, consistent with multiple independent introductions from regional *C. auris* followed by within-unit spread.

### Integrated Genomic and Epidemiologic Investigation of Outbreak Clusters

Clusters 2 and 4 contained exclusively BICU isolates (Figure 3, panels C, E), whereas clusters 1 and 3 contained sequences collected within the medical center but outside of the BICU (Figure 3, panels B, D). In one instance, in cluster 1 (Figure 3, panel B), 1 sequence from the same facility collected 4 months before the first BICU case was identical to the first BICU case’s genome (patient 1). No epidemiologic links to BICU patients in this cluster were identified, and facility stays were separated by 123 days. In cluster 3 (Figure 3, panel D), 1 sequence from another unit within the facility and 1 sequence from elsewhere in the medical system collected 1 month before and 1 month after the first BICU case clustered with BICU isolates (patients 14, 15, 16, 18). No epidemiologic links were identified between the cases from the same facility. The case-patient from elsewhere in the medical system had been hospitalized at the BICU facility the month before, overlapping with other patients in this cluster. In addition, this patient and another BICU patient with *C. auris* from this cluster were both exposed to the same healthcare worker (speech therapist) during overlapping timeframes. Contextual isolates from institutions outside of the medical system did not fall in BICU clusters (>8 SNPs) (Figure 3; Appendix Figure 1).

BICU sequences clustered by collection date; clusters generally contained isolates collected within 3–6 months of each other (Figure 3, panels B–E). Clustering patients had overlapping BICU admission dates in all but 1 instance (Figure 2). In the exception, a patient (patient 9) from cluster 1 was admitted to the BICU 91 days after other patients in cluster 1 were discharged, but the *C. auris* isolate was not sequenced. This patient was then discharged to the same long-term acute care hospital as another patient in cluster 1 (Figure 3, panel B). The isolate belonging to cluster 1 was identified in an admission screening culture 8 months later, when the patient was admitted to another unit within the BICU facility. Thus, *C. auris* transmission may have occurred either on the BICU or at the long-term acute care hospital.

### Analysis of Longitudinal Outbreak Isolates

Longitudinal isolates were collected from 3 patients over 51 (n = 2), 63 (n = 6), and 135 (n = 3) days and included both clinical and screening isolates. All longitudinal sequences clustered closely with other sequences from the same patient. Six of 11 longitudinal sequences were identical to another sequence from the same person; clinical and screening isolates were often identical to one another (Figure 3, panels C–E). Specimens collected from the same person had a mean 1.2 (range 1–4) SNP differences (Figure 4).

We investigated mutations in antifungal resistance–associated genes to look for longitudinal acquisition of antifungal resistance mutations. We identified a mutation associated with azole resistance in the *TAC1b* gene (I187T) in the last isolate collected from 1 patient; the 2 isolates obtained from this person earlier did not contain this mutation (Figure 3, panel D) (*32**,**33*). The patient received voriconazole therapy after collection of the isolates lacking the mutation but before the emergence of the *TAC1b* mutation. The isolate was not subjected to phenotypic antifungal susceptibility testing.

## Discussion

We describe a *C. auris* outbreak in a BICU that resulted in 28 patients colonized or infected over 2 years. We initially hypothesized that this was one continuous outbreak with possible environmental reservoirs on the unit contributing to ongoing transmission. WGS revealed 4 distinct clusters and 7 distinct genotypes; integration with epidemiologic information identified a complex outbreak that was driven both by importation of new strains and by within-BICU cross-transmission. Two phylogenetic clusters that included 7 (32%) of the sequenced case patient isolates contained isolates from both BICU patients and patients who were cared for in other units within the medical center, suggesting that *C. auris* might have been imported into the BICU from elsewhere in the facility by contaminated healthcare provider hands or clothing or by shared equipment. Alternatively, a BICU patient might have acquired *C. auris* upon exposure to contaminated surfaces or equipment in a common diagnostic or procedure area outside of the BICU. Either of those pathways could have led to the index BICU case; the index isolate genome was identical to an isolate collected from a patient in a unit elsewhere in the medical center 4 months earlier. However, epidemiologic links between new case-patients on the BICU and other patients in the hospital were not always identified.

Occult colonization, or colonization at low level or unsampled sites that results in nondetection by surveillance culture, of newly admitted patients might also have contributed to importation of *C. auris* into the BICU. Although all patients underwent axilla or groin screening for *C. auris* at the time of BICU admission, the sensitivity of this approach has been reported at ≈62%; to detect 100% of colonized patients, >6 body sites needed to be screened (*15*). Indeed, 3 patients were colonized with unique isolates that did not fall into any of the 4 clusters. Further, 11 of 22 case-patients whose isolates were sequenced and who had temporally overlapping BICU stays were included in 2 clusters that included only BICU patient isolates, suggesting within-BICU transmission of *C. auris*. Thus, undetected colonization at the time of admission and infection control breaches likely enabled introduction and transmission of *C. auris* to occur on the BICU. As our study demonstrated, *C. auris* is transmitted easily in healthcare facilities, and regional transmission can be hastened by patient transfers; in this outbreak, 61% of colonized or infected patients were discharged to other healthcare facilities. Interfacility communication and strict infection control measures are necessary to limit spread to other patient populations.

Once *C. auris* was introduced on the BICU, transmission was likely exacerbated by observed infection control breaches, particularly poor hand hygiene practices and lapses in cleaning of shared equipment. The prolonged lengths of stay of the patients (mean 67 days) also pose infection prevention and control challenges; *C. auris* rooms are recontaminated in as little as 4 hours after disinfection (*34*), emphasizing the need for stringent long-term adherence to cleaning and basic infection control practices.

The first limitation of our study is that it was conducted retrospectively; we selected samples for WGS on the basis of availability of stored isolates, and not all isolates from the outbreak were sequenced. Second, in most cases, only 1 isolate per patient was available for sequencing. Although some studies have found that patients can carry multiple genetically distinct *C. auris* isolates, genetically similar isolates may be more likely in an acute outbreak setting (*9*,*21*). In our study, all isolates collected from the same patient were closely related. Third, collection of epidemiologic metadata was limited to medical record review; some activities would not be recorded in the medical record. Further, contamination of portable unit-based equipment that is shared between patients might have contributed to ongoing *C. auris* transmission, but this possibility could not be verified through medical record review, and we conducted no environmental culturing.

WGS refined our understanding of this *C. auris* outbreak. The discovery that the outbreak included multiple introductions of *C. auris* onto the unit influenced our current approach to *C. auris* investigation and response; we focus now on between-unit transmission, including the possible role of ancillary personnel who move throughout the hospital, and not just on within-unit infection prevention measures. Furthermore, we conduct admission screening as well as point prevalence survey protocols in response to a *C. auris* case to identify and isolate colonized patients quickly. Integrated WGS and epidemiologic investigation is a powerful tool for identifying drivers of transmission in nosocomial outbreaks.

Appendix Additional information about *Candida auris* outbreak in burn intensive care unit, Illinois, USA, 2021–2023.
