# Supplementary material for: Candida auris Outbreak and Epidemiologic Response in Burn Intensive Care Unit, Illinois, USA, 2021–2023
Source: Emerg Infect Dis. 2025 Mar;31(3):438–47. doi: 10.3201/eid3103.241195 (PMC11878305; doi:10.3201/eid3103.241195)
Supplement: Appendix — Additional information about Candida auris outbreak in burn intensive care unit, Illinois, USA, 2021–2023. [file 24-1195-Techapp-s1.pdf]

Article DOI: https://doi.org/10.3201/eid3103.241195

EID cannot ensure accessibility for supplementary materials supplied by authors. Readers who have difficulty accessing supplementary content should contact the authors for assistance.

Candida auris Outbreak and Epidemiologic Response in a Burn Intensive Care Unit, Illinois, United States, 2021–2023

Appendix

Appendix Table. *Candida auris* sequences from study of outbreak in burn intensive care unit, Illinois, United States, 2021–2023

SRA accession	Specimen source	Figure 3	Appendix Figure 1
SRR27957377	BICU	Included	Included
SRR27957376	BICU	Included	Included
SRR27957375	BICU	Included	Included
SRR27957374	BICU	Included	Included
SRR27957373	BICU	Included	Included
SRR27957372	BICU	Included	Included
SRR27957370	BICU	Included	Included
SRR27957369	BICU	Included	Included
SRR27957368	BICU	Included	Included
SRR27957367	BICU	Included	Included
SRR22393499	BICU	Included	Included
SRR22393498	BICU	Included	Included
SRR22393497	BICU	Included	Included
SRR22393496	BICU	Included	Included
SRR24673435	BICU	Included	Included
SRR24673434	BICU	Included	Included
SRR24673423	BICU	Included	Included
SRR24673412	BICU	Included	Included
SRR24673401	BICU	Included	Included
SRR24673390	BICU	Included	Included
SRR24673379	BICU	Included	Included
SRR24673377	BICU	Included	Included
SRR24673376	BICU	Included	Included
SRR24673385	BICU	Included	Included
SRR24673384	BICU	Included	Included
SRR24673383	BICU	Included	Included
SRR24673382	BICU	Included	Included
SRR24673381	BICU	Included	Included
SRR24673418	Medical system	Included	Included
SRR24673417	Medical system	Included	Included
SRR24673416	Medical system	Included	Included
SRR24673415	Medical system	Included	Included
SRR24673414	Medical system	Included	Included
SRR24673413	Medical system	Included	Included
SRR24673411	Medical system	Included	Included
SRR24673410	Medical system	Included	Included
SRR24673409	Medical system	Included	Included
SRR24673408	Medical system	Included	Included
SRR24673407	Medical system	Included	Included
SRR24673406	Medical system	Included	Included
SRR24673405	Medical system	Included	Included
SRR24673404	Medical system	Included	Included
SRR24673403	Medical system	Included	Included

SRA accession	Specimen source	Figure 3	Appendix Figure 1
SRR24673402	Medical system	Included	Included
SRR24673400	Medical system	Included	Included
SRR24673399	Medical system	Included	Included
SRR24673398	Medical system	Included	Included
SRR24673397	Medical system	Included	Included
SRR24673396	Medical system	Included	Included
SRR24673395	Medical system	Included	Included
SRR24673394	Medical system	Included	Included
SRR24673393	Medical system	Included	Included
SRR24673392	Medical system	Included	Included
SRR24673391	Medical system	Included	Included
SRR24673389	Medical system	Included	Included
SRR24673388	Medical system	Included	Included
SRR24673387	Medical system	Included	Included
SRR24673386	Medical system	Included	Included
SRR22393500	Same facility	Included	Included
SRR22393495	Same facility	Included	Included
SRR24673375	Same facility	Included	Included
SRR24673433	Same facility	Included	Included
SRR24673432	Same facility	Included	Included
SRR24673431	Same facility	Included	Included
SRR24673430	Same facility	Included	Included
SRR24673429	Same facility	Included	Included
SRR24673428	Same facility	Included	Included
SRR24673427	Same facility	Included	Included
SRR24673426	Same facility	Included	Included
SRR24673425	Same facility	Included	Included
SRR24673424	Same facility	Included	Included
SRR24673422	Same facility	Included	Included
SRR24673421	Same facility	Included	Included
SRR24673420	Same facility	Included	Included
SRR24673419	Same facility	Included	Included
SRR24673380	Same facility	Included	Included
SRR24673378	Same facility	Included	Included
SRR7909141	Illinois contextual	Not_included	Included
SRR7909220	Illinois contextual	Not_included	Included
SRR7909238	Illinois contextual	Not_included	Included
SRR7909309	Illinois contextual	Not_included	Included
SRR7909391	Illinois contextual	Not_included	Included
SRR7909250	Illinois contextual	Not_included	Included
SRR7909191	Illinois contextual	Not_included	Included
SRR7909407	Illinois contextual	Not_included	Included
SRR7909256	Illinois contextual	Not_included	Included
SRR7909248	Illinois contextual	Not_included	Included
SRR7909228	Illinois contextual	Not_included	Included
SRR7909172	Illinois contextual	Not_included	Included
SRR7909387	Illinois contextual	Not_included	Included
SRR7909179	Illinois contextual	Not_included	Included
SRR7909388	Illinois contextual	Not_included	Included
SRR7909335	Illinois contextual	Not_included	Included
SRR7909151	Illinois contextual	Not_included	Included
SRR7909366	Illinois contextual	Not_included	Included
SRR7909138	Illinois contextual	Not_included	Included
SRR7909158	Illinois contextual	Not_included	Included
SRR7909245	Illinois contextual	Not_included	Included
SRR7909200	Illinois contextual	Not_included	Included
SRR7909145	Illinois contextual	Not_included	Included
SRR7909294	Illinois contextual	Not_included	Included
SRR7909221	Illinois contextual	Not_included	Included
SRR7909405	Illinois contextual	Not_included	Included
SRR7909227	Illinois contextual	Not_included	Included
SRR7909315	Illinois contextual	Not_included	Included
SRR7909226	Illinois contextual	Not_included	Included
SRR7909357	Illinois contextual	Not_included	Included
SRR23919790	Illinois contextual	Included	Included
SRR23919789	Illinois contextual	Included	Included
SRR23919786	Illinois contextual	Included	Included
SRR23919785	Illinois contextual	Included	Included
SRR23919784	Illinois contextual	Included	Included

SRA accession	Specimen source	Figure 3	Appendix Figure 1
SRR23919783	Illinois contextual	Included	Included
SRR23919782	Illinois contextual	Included	Included
SRR23919781	Illinois contextual	Included	Included
SRR23919780	Illinois contextual	Included	Included
SRR23919779	Illinois contextual	Included	Included
SRR23919788	Illinois contextual	Included	Included
SRR23919787	Illinois contextual	Included	Included
SRR12363130	Illinois contextual	Not_included	Included
SRR12363129	Illinois contextual	Not_included	Included
SRR12363163	Illinois contextual	Not_included	Included
SRR12363162	Illinois contextual	Not_included	Included
SRR12363161	Illinois contextual	Not_included	Included
SRR12363160	Illinois contextual	Not_included	Included
SRR12363159	Illinois contextual	Not_included	Included
SRR12363158	Illinois contextual	Not_included	Included
SRR12363157	Illinois contextual	Not_included	Included
SRR12363156	Illinois contextual	Not_included	Included
SRR12363155	Illinois contextual	Not_included	Included
SRR12363154	Illinois contextual	Not_included	Included
SRR12363152	Illinois contextual	Not_included	Included
SRR12363151	Illinois contextual	Not_included	Included
SRR12363150	Illinois contextual	Not_included	Included
SRR12363149	Illinois contextual	Not_included	Included
SRR12363148	Illinois contextual	Not_included	Included
SRR12363147	Illinois contextual	Not_included	Included
SRR12363146	Illinois contextual	Not_included	Included
SRR12363145	Illinois contextual	Not_included	Included
SRR12363144	Illinois contextual	Not_included	Included
SRR12363143	Illinois contextual	Not_included	Included
SRR12363141	Illinois contextual	Not_included	Included
SRR12363140	Illinois contextual	Not_included	Included
SRR12363139	Illinois contextual	Not_included	Included
SRR27223619	Illinois contextual	Included	Included
SRR27223618	Illinois contextual	Included	Included
SRR27223617	Illinois contextual	Included	Included
SRR27223616	Illinois contextual	Included	Included
SRR27223615	Illinois contextual	Included	Included
SRR27223614	Illinois contextual	Included	Included
SRR27957383	Illinois contextual	Included	Included
SRR27957382	Illinois contextual	Included	Included
SRR27957381	Illinois contextual	Included	Included
SRR27957380	Illinois contextual	Included	Included
SRR27957379	Illinois contextual	Included	Included
SRR27957378	Illinois contextual	Included	Included
SRR27957371	Illinois contextual	Included	Included
SRR27957366	Illinois contextual	Included	Included
SRR27957364	Illinois contextual	Included	Included
SRR27957365	Illinois contextual	Included	Included
SRR27957363	Illinois contextual	Included	Included
SRR27957362	Illinois contextual	Included	Included
SRR27957361	Illinois contextual	Included	Included
SRR27957360	Illinois contextual	Included	Included
SRR27957359	Illinois contextual	Included	Included
SRR27957358	Illinois contextual	Included	Included
SRR27223671	Illinois contextual	Included	Included
SRR27223670	Illinois contextual	Included	Included
SRR27223669	Illinois contextual	Included	Included
SRR27223668	Illinois contextual	Included	Included
SRR27223667	Illinois contextual	Included	Included
SRR27223666	Illinois contextual	Included	Included
SRR27223620	Illinois contextual	Included	Included
SRR27223664	Illinois contextual	Included	Included
SRR27223663	Illinois contextual	Included	Included
SRR27223802	Illinois contextual	Not_included	Included
SRR27223801	Illinois contextual	Not_included	Included
SRR27223572	Illinois contextual	Not_included	Included
SRR27223611	Illinois contextual	Not_included	Included
SRR27223600	Illinois contextual	Not_included	Included
SRR27223589	Illinois contextual	Not_included	Included

SRA accession	Specimen source	Figure 3	Appendix Figure 1
SRR27223546	Illinois contextual	Not_included	Included
SRR27223535	Illinois contextual	Not_included	Included
SRR27223738	Illinois contextual	Not_included	Included
SRR27223727	Illinois contextual	Not_included	Included
SRR27223662	Illinois contextual	Not_included	Included
SRR27223800	Illinois contextual	Not_included	Included
SRR27223789	Illinois contextual	Not_included	Included
SRR27223778	Illinois contextual	Not_included	Included
SRR27223713	Illinois contextual	Not_included	Included
SRR27223702	Illinois contextual	Not_included	Included
SRR27223691	Illinois contextual	Not_included	Included
SRR27223648	Illinois contextual	Not_included	Included
SRR27223637	Illinois contextual	Not_included	Included
SRR27223626	Illinois contextual	Not_included	Included
SRR27223583	Illinois contextual	Not_included	Included
SRR27223571	Illinois contextual	Not_included	Included
SRR27223560	Illinois contextual	Not_included	Included
SRR27223763	Illinois contextual	Not_included	Included
SRR27223752	Illinois contextual	Not_included	Included
SRR27223741	Illinois contextual	Not_included	Included
SRR27223676	Illinois contextual	Not_included	Included
SRR27223665	Illinois contextual	Not_included	Included
SRR27223654	Illinois contextual	Not_included	Included
SRR27223661	Illinois contextual	Not_included	Included
SRR27223613	Illinois contextual	Not_included	Included
SRR27223612	Illinois contextual	Not_included	Included
SRR27223610	Illinois contextual	Not_included	Included
SRR27223609	Illinois contextual	Not_included	Included
SRR27223608	Illinois contextual	Not_included	Included
SRR27223607	Illinois contextual	Not_included	Included
SRR27223606	Illinois contextual	Not_included	Included
SRR27223605	Illinois contextual	Not_included	Included
SRR27223604	Illinois contextual	Not_included	Included
SRR27223603	Illinois contextual	Not_included	Included
SRR27223602	Illinois contextual	Not_included	Included
SRR27223601	Illinois contextual	Not_included	Included
SRR27223599	Illinois contextual	Not_included	Included
SRR27223598	Illinois contextual	Not_included	Included
SRR27223597	Illinois contextual	Not_included	Included
SRR27223596	Illinois contextual	Not_included	Included
SRR27223595	Illinois contextual	Not_included	Included
SRR27223594	Illinois contextual	Not_included	Included
SRR27223593	Illinois contextual	Not_included	Included
SRR27223592	Illinois contextual	Not_included	Included
SRR27223591	Illinois contextual	Not_included	Included
SRR27223590	Illinois contextual	Not_included	Included
SRR27223556	Illinois contextual	Not_included	Included
SRR27223555	Illinois contextual	Not_included	Included
SRR27223554	Illinois contextual	Not_included	Included
SRR27223553	Illinois contextual	Not_included	Included
SRR27223552	Illinois contextual	Not_included	Included
SRR27223551	Illinois contextual	Not_included	Included
SRR27223550	Illinois contextual	Not_included	Included
SRR27223549	Illinois contextual	Not_included	Included
SRR27223548	Illinois contextual	Not_included	Included
SRR27223547	Illinois contextual	Not_included	Included
SRR27223545	Illinois contextual	Not_included	Included
SRR27223544	Illinois contextual	Not_included	Included
SRR27223543	Illinois contextual	Not_included	Included
SRR27223542	Illinois contextual	Not_included	Included
SRR27223541	Illinois contextual	Not_included	Included
SRR27223540	Illinois contextual	Not_included	Included
SRR27223539	Illinois contextual	Not_included	Included
SRR27223538	Illinois contextual	Not_included	Included
SRR27223537	Illinois contextual	Not_included	Included
SRR27223536	Illinois contextual	Not_included	Included
SRR27223534	Illinois contextual	Not_included	Included
SRR27223533	Illinois contextual	Not_included	Included
SRR27223532	Illinois contextual	Not_included	Included

SRA accession	Specimen source	Figure 3	Appendix Figure 1
SRR27223531	Illinois contextual	Not_included	Included
SRR27223530	Illinois contextual	Not_included	Included
SRR27223529	Illinois contextual	Not_included	Included
SRR27223528	Illinois contextual	Not_included	Included
SRR27223527	Illinois contextual	Not_included	Included
SRR27223526	Illinois contextual	Not_included	Included
SRR27223525	Illinois contextual	Not_included	Included
SRR27223737	Illinois contextual	Not_included	Included
SRR27223736	Illinois contextual	Not_included	Included
SRR27223660	Illinois contextual	Not_included	Included
SRR27223735	Illinois contextual	Not_included	Included
SRR27223734	Illinois contextual	Not_included	Included
SRR27223733	Illinois contextual	Not_included	Included
SRR27223732	Illinois contextual	Not_included	Included
SRR27223731	Illinois contextual	Not_included	Included
SRR27223730	Illinois contextual	Not_included	Included
SRR27223729	Illinois contextual	Not_included	Included
SRR27223728	Illinois contextual	Not_included	Included
SRR27223726	Illinois contextual	Not_included	Included
SRR27223725	Illinois contextual	Not_included	Included
SRR27223724	Illinois contextual	Not_included	Included
SRR27223723	Illinois contextual	Not_included	Included
SRR27223722	Illinois contextual	Not_included	Included
SRR27223721	Illinois contextual	Not_included	Included
SRR27223720	Illinois contextual	Not_included	Included
SRR27223719	Illinois contextual	Not_included	Included
SRR27223718	Illinois contextual	Not_included	Included
SRR27223717	Illinois contextual	Not_included	Included
SRR27223799	Illinois contextual	Not_included	Included
SRR27223798	Illinois contextual	Not_included	Included
SRR27223797	Illinois contextual	Not_included	Included
SRR27223796	Illinois contextual	Not_included	Included
SRR27223795	Illinois contextual	Not_included	Included
SRR27223794	Illinois contextual	Not_included	Included
SRR27223793	Illinois contextual	Not_included	Included
SRR27223792	Illinois contextual	Not_included	Included
SRR27223791	Illinois contextual	Not_included	Included
SRR27223790	Illinois contextual	Not_included	Included
SRR27223788	Illinois contextual	Not_included	Included
SRR27223787	Illinois contextual	Not_included	Included
SRR27223786	Illinois contextual	Not_included	Included
SRR27223785	Illinois contextual	Not_included	Included
SRR27223784	Illinois contextual	Not_included	Included
SRR27223783	Illinois contextual	Not_included	Included
SRR27223782	Illinois contextual	Not_included	Included
SRR27223781	Illinois contextual	Not_included	Included
SRR27223780	Illinois contextual	Not_included	Included
SRR27223779	Illinois contextual	Not_included	Included
SRR27223777	Illinois contextual	Not_included	Included
SRR27223776	Illinois contextual	Not_included	Included
SRR27223775	Illinois contextual	Not_included	Included
SRR27223774	Illinois contextual	Not_included	Included
SRR27223773	Illinois contextual	Not_included	Included
SRR27223772	Illinois contextual	Not_included	Included
SRR27223771	Illinois contextual	Not_included	Included
SRR27223716	Illinois contextual	Not_included	Included
SRR27223715	Illinois contextual	Not_included	Included
SRR27223714	Illinois contextual	Not_included	Included
SRR27223712	Illinois contextual	Not_included	Included
SRR27223711	Illinois contextual	Not_included	Included
SRR27223710	Illinois contextual	Not_included	Included
SRR27223659	Illinois contextual	Not_included	Included
SRR27223709	Illinois contextual	Not_included	Included
SRR27223708	Illinois contextual	Not_included	Included
SRR27223707	Illinois contextual	Not_included	Included
SRR27223706	Illinois contextual	Not_included	Included
SRR27223705	Illinois contextual	Not_included	Included
SRR27223704	Illinois contextual	Not_included	Included
SRR27223703	Illinois contextual	Not_included	Included

SRA accession	Specimen source	Figure 3	Appendix Figure 1
SRR27223701	Illinois contextual	Not_included	Included
SRR27223700	Illinois contextual	Not_included	Included
SRR27223699	Illinois contextual	Not_included	Included
SRR27223698	Illinois contextual	Not_included	Included
SRR27223697	Illinois contextual	Not_included	Included
SRR27223696	Illinois contextual	Not_included	Included
SRR27223695	Illinois contextual	Not_included	Included
SRR27223694	Illinois contextual	Not_included	Included
SRR27223693	Illinois contextual	Not_included	Included
SRR27223692	Illinois contextual	Not_included	Included
SRR27223690	Illinois contextual	Not_included	Included
SRR27223689	Illinois contextual	Not_included	Included
SRR27223688	Illinois contextual	Not_included	Included
SRR27223687	Illinois contextual	Not_included	Included
SRR27223686	Illinois contextual	Not_included	Included
SRR27223685	Illinois contextual	Not_included	Included
SRR27223652	Illinois contextual	Not_included	Included
SRR27223651	Illinois contextual	Not_included	Included
SRR27223650	Illinois contextual	Not_included	Included
SRR27223649	Illinois contextual	Not_included	Included
SRR27223647	Illinois contextual	Not_included	Included
SRR27223646	Illinois contextual	Not_included	Included
SRR27223645	Illinois contextual	Not_included	Included
SRR27223644	Illinois contextual	Not_included	Included
SRR27223643	Illinois contextual	Not_included	Included
SRR27223642	Illinois contextual	Not_included	Included
SRR27223641	Illinois contextual	Not_included	Included
SRR27223640	Illinois contextual	Not_included	Included
SRR27223639	Illinois contextual	Not_included	Included
SRR27223638	Illinois contextual	Not_included	Included
SRR27223636	Illinois contextual	Not_included	Included
SRR27223635	Illinois contextual	Not_included	Included
SRR27223634	Illinois contextual	Not_included	Included
SRR27223633	Illinois contextual	Not_included	Included
SRR27223632	Illinois contextual	Not_included	Included
SRR27223631	Illinois contextual	Not_included	Included
SRR27223630	Illinois contextual	Not_included	Included
SRR27223629	Illinois contextual	Not_included	Included
SRR27223628	Illinois contextual	Not_included	Included
SRR27223627	Illinois contextual	Not_included	Included
SRR27223625	Illinois contextual	Not_included	Included
SRR27223624	Illinois contextual	Not_included	Included
SRR27223623	Illinois contextual	Not_included	Included
SRR27223622	Illinois contextual	Not_included	Included
SRR27223621	Illinois contextual	Not_included	Included
SRR27223588	Illinois contextual	Not_included	Included
SRR27223587	Illinois contextual	Not_included	Included
SRR27223586	Illinois contextual	Not_included	Included
SRR27223585	Illinois contextual	Not_included	Included
SRR27223584	Illinois contextual	Not_included	Included
SRR27223582	Illinois contextual	Not_included	Included
SRR27223581	Illinois contextual	Not_included	Included
SRR27223580	Illinois contextual	Not_included	Included
SRR27223579	Illinois contextual	Not_included	Included
SRR27223578	Illinois contextual	Not_included	Included
SRR27223577	Illinois contextual	Not_included	Included
SRR27223576	Illinois contextual	Not_included	Included
SRR27223575	Illinois contextual	Not_included	Included
SRR27223574	Illinois contextual	Not_included	Included
SRR27223573	Illinois contextual	Not_included	Included
SRR27223570	Illinois contextual	Not_included	Included
SRR27223569	Illinois contextual	Not_included	Included
SRR27223568	Illinois contextual	Not_included	Included
SRR27223567	Illinois contextual	Not_included	Included
SRR27223566	Illinois contextual	Not_included	Included
SRR27223565	Illinois contextual	Not_included	Included
SRR27223564	Illinois contextual	Not_included	Included
SRR27223563	Illinois contextual	Not_included	Included
SRR27223562	Illinois contextual	Not_included	Included

SRA accession	Specimen source	Figure 3	Appendix Figure 1
SRR27223561	Illinois contextual	Not_included	Included
SRR27223559	Illinois contextual	Not_included	Included
SRR27223558	Illinois contextual	Not_included	Included
SRR27223557	Illinois contextual	Not_included	Included
SRR27223770	Illinois contextual	Not_included	Included
SRR27223769	Illinois contextual	Not_included	Included
SRR27223768	Illinois contextual	Not_included	Included
SRR27223767	Illinois contextual	Not_included	Included
SRR27223766	Illinois contextual	Not_included	Included
SRR27223765	Illinois contextual	Not_included	Included
SRR27223764	Illinois contextual	Not_included	Included
SRR27223762	Illinois contextual	Not_included	Included
SRR27223761	Illinois contextual	Not_included	Included
SRR27223760	Illinois contextual	Not_included	Included
SRR27223759	Illinois contextual	Not_included	Included
SRR27223758	Illinois contextual	Not_included	Included
SRR27223757	Illinois contextual	Not_included	Included
SRR27223756	Illinois contextual	Not_included	Included
SRR27223755	Illinois contextual	Not_included	Included
SRR27223754	Illinois contextual	Not_included	Included
SRR27223754	Illinois contextual	Not_included	Included
SRR27223753	Illinois contextual	Not_included	Included
SRR27223751	Illinois contextual	Not_included	Included
SRR27223750	Illinois contextual	Not_included	Included
SRR27223749	Illinois contextual	Not_included	Included
SRR27223748	Illinois contextual	Not_included	Included
SRR27223657	Illinois contextual	Not_included	Included
SRR27223747	Illinois contextual	Not_included	Included
SRR27223746	Illinois contextual	Not_included	Included
SRR27223745	Illinois contextual	Not_included	Included
SRR27223744	Illinois contextual	Not_included	Included
SRR27223743	Illinois contextual	Not_included	Included
SRR27223742	Illinois contextual	Not_included	Included
SRR27223740	Illinois contextual	Not_included	Included
SRR27223656	Illinois contextual	Not_included	Included
SRR27223655	Illinois contextual	Not_included	Included
SRR27223739	Illinois contextual	Not_included	Included
SRR27223684	Illinois contextual	Not_included	Included
SRR27223683	Illinois contextual	Not_included	Included
SRR27223682	Illinois contextual	Not_included	Included
SRR27223681	Illinois contextual	Not_included	Included
SRR27223653	Illinois contextual	Not_included	Included
SRR27223680	Illinois contextual	Not_included	Included
SRR27223679	Illinois contextual	Not_included	Included
SRR27223678	Illinois contextual	Not_included	Included
SRR27223677	Illinois contextual	Not_included	Included
SRR27223675	Illinois contextual	Not_included	Included
SRR27223674	Illinois contextual	Not_included	Included
SRR27223673	Illinois contextual	Not_included	Included
SRR27223672	Illinois contextual	Not_included	Included

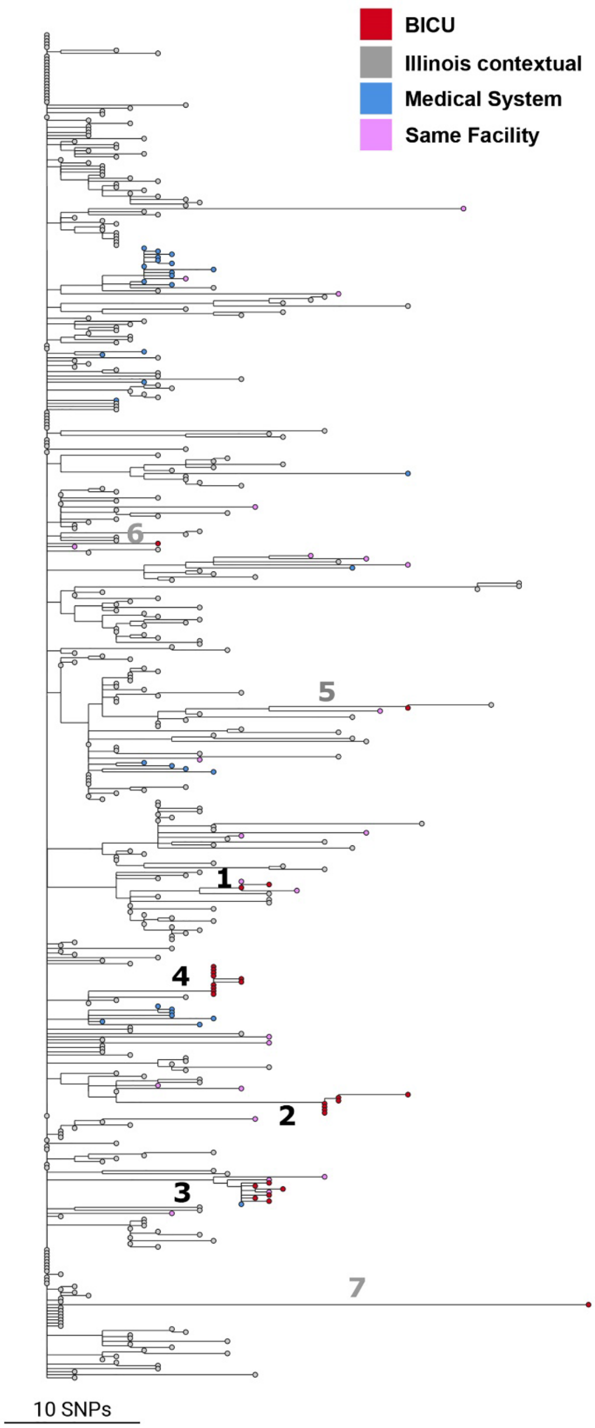

Appendix Figure. Neighbor-joining SNP-based phylogenetic tree of sequences from BICU isolates (red), isolates collected from the same facility (pink) or another facility within the medical system (blue) and all publicly available Illinois sequences available as of April 2024 (gray) (Appendix Table 1). Facility source for 314 of 361 Illinois contextual sequences was confirmed not to be within the BICU medical system. The facility source of the remaining 47 of 361 isolate sequences was not known. Branch lengths are SNP distances. SNP, single-nucleotide polymorphism.
